# Automated Reconstruction Algorithm for Identification of 3D Architectures of Cribriform Ductal Carcinoma *In Situ*

**DOI:** 10.1371/journal.pone.0044011

**Published:** 2012-09-06

**Authors:** Kerri-Ann Norton, Sameera Namazi, Nicola Barnard, Mariko Fujibayashi, Gyan Bhanot, Shridar Ganesan, Hitoshi Iyatomi, Koichi Ogawa, Troy Shinbrot

**Affiliations:** 1 Biomedical Engineering, Rutgers University, Piscataway, New Jersey, United States of America; 2 BioMaPS Institute, Rutgers University, Piscataway, New Jersey, United States of America; 3 Department of Biomedical Engineering, School of Medicine, Johns Hopkins University, Baltimore, Maryland, United States of America; 4 Chemical Engineering, Rutgers University, Piscataway, New Jersey, United States of America; 5 Department of Pathology, UMDNJ-Robert Wood Johnson Medical School, New Brunswick, New Jersey, United States of America; 6 Department of Surgical Pathology, Tokyo Women's Medical University, Medical Center East, Tokyo, Japan; 7 Simons Center for Systems Biology, Institute for Advanced Study, Princeton, New Jersey, United States of America; 8 Cancer Institute of New Jersey, UMDNJ-Robert Wood Johnson Medical School, New Brunswick, New Jersey, United States of America; 9 Department of Applied Informatics, Hosei University, Tokyo, Japan; University Medical Centre Utrecht, The Netherlands

## Abstract

Ductal carcinoma in situ (DCIS) is a pre-invasive carcinoma of the breast that exhibits several distinct morphologies but the link between morphology and patient outcome is not clear. We hypothesize that different mechanisms of growth may still result in similar 2D morphologies, which may look different in 3D. To elucidate the connection between growth and 3D morphology, we reconstruct the 3D architecture of cribriform DCIS from resected patient material. We produce a fully automated algorithm that aligns, segments, and reconstructs 3D architectures from microscopy images of 2D serial sections from human specimens. The alignment algorithm is based on normalized cross correlation, the segmentation algorithm uses histogram equilization, Otsu's thresholding, and morphology techniques to segment the duct and cribra. The reconstruction method combines these images in 3D. We show that two distinct 3D architectures are indeed found in samples whose 2D histological sections are similarly identified as cribriform DCIS. These differences in architecture support the hypothesis that luminal spaces may form due to different mechanisms, either isolated cell death or merging fronds, leading to the different architectures. We find that out of 15 samples, 6 were found to have ‘bubble-like’ cribra, 6 were found to have ‘tube-like’ criba and 3 were ‘unknown.’ We propose that the 3D architectures found, ‘bubbles’ and ‘tubes’, account for some of the heterogeneity of the disease and may be prognostic indicators of different patient outcomes.

## Introduction

Ductal carcinoma in situ (DCIS) is classified and graded by pathologists using 2-dimensional (2D) histological cross-sections of biopsy specimens. Unfortunately, actual correlations between 2D morphology and patient outcome are not strong [1], [2]. In a study that compared multiple specimens within a duct and multiple ducts within patients with DCIS, it was found that patients with DCIS exhibit significant heterogeneity [3]. Alongside this state of affairs, experimental [4], [5] and theoretical [6] studies have reported that growth patterns of breast tissue differ significantly in 2D and in 3D. Extensive *in vitro* analyses in particular have demonstrated that cancer growth behaviors [7]–[9] and architectures [10]–[13] are qualitatively different in 2D and 3D, and several computational models of DCIS have consequently been developed to investigate both 2D and 3D characteristics of DCIS development [14]–[20].

3D reconstruction techniques have been used in many biological systems, ranging from ascidian morphogenesis to virtual colonoscopy to facial reconstruction [21]–[24]. An early review of 3D techniques used to study 3D organization and structure of DCIS appears in [25], and a particularly germane investigation examining the 3D ductal structure of a normal breast can be found in [26]. In addition, other imaging techniques have been useful for studying architecture and prognosis in prostate [27], [28], colorectal [29], and ductal carcinoma *in situ* (DCIS) tissues [30]. These studies of DCIS provide a compelling case that 3D reconstructions are capable of advancing the understanding of the structure of cancers in general, and DCIS in particular. 3D reconstructions of DCIS have nevertheless been limited to date [26], [31]–[34], and focus (1) on measuring the extent of the disease through the mammary system at comparatively low resolution (i.e. down to about 1 mm), or (2) on identifying sites of origin of DCIS.

Studies of the extent of DCIS include the use of a system for morphological and molecular analysis of thick tissue, for which DCIS is one particular example [35]. Additionally, x-ray computed tomography (CT) has been used as a tool to visualize DCIS, with attention given to areas of micro-calcification that can be well identified using this technique [36]. Calcifications in a high-grade specimen of DCIS have been identified [37], supporting the conclusion that DCIS was continuous through the duct [37], as proposed also in earlier research [32]. Other studies have examined the distribution of DCIS, but only in cases with infiltrating carcinoma (IC), leading to the finding that DCIS spreads in a fan-like geometry [38].

3D studies of DCIS focusing on identifying sites of origin include work using a quantitative subgross method to examine DCIS specimens [34]. Wellings et al. reported that DCIS in the 60 specimens tested originated most frequently in terminal or lobule units [34]. Another study used reconstructions to determine that there is a relationship between the presence of carcinoma in papillomas and the origin of the carcinomas [33]. 3D reconstructions of DCIS from biopsy specimens have also been used to identify the outer margins of the disease, which is of manifest importance for excisions [39].

Notwithstanding these important studies, analysis of potential differences between 2D and 3D morphologies of DCIS at the micro-scale (down to microns) that we focus on here do not appear in the literature. In particular, we hypothesize that there may be distinct 3D morphologies of DCIS that can be identified using microscopy images of serially sectioned resected human specimens. Additionally, these 3D morphologies may be produced through different growth mechanisms, and may appear to be morphologically similar in 2D sections.

**Figure 1:**
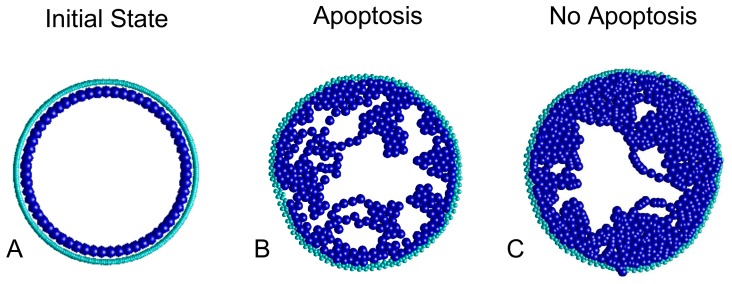
Simulations of DCIS development. **A** The initial duct starting from the original state of reproducing epithelial cells (blue) surrounded by myoepithelial sheath (cyan). Cribriform subtype is produced at **B** high reproductive rate with apoptosis, and **C** high reproductive rate without apoptosis (for simulation details, see: Norton et al., 2010).

Our motivation for this hypothesis is illustrated in Fig. 1, where we show two different cribriform morphologies produced from *in silico* simulations [40] of DCIS growth. In these simulations, a monolayer (Fig. 1A) of polar epithelial cells (blue) surrounded by a sheath of myoepithelial cells (cyan) are permitted to mitose, migrate, and apoptose at prescribed rates. Cells interact biomechanically, using a “Voigt,” damped-spring model, and all cells exhibit cohesion to a left- and right- neighbor, which maintains their polarization, so that the apical side of all epithelial cells always point toward the luminal space. Details appear in [40].

From these simulations we find that a cribriform subtype appears - due to very different causes - either with (Fig. 1B) or without (Fig. 1C) apoptosis. To simulate apoptosis, we remove cells from the simulation when they are overcrowded (defined to occur when they acquire more than 10 neighbors), and we reconnect their left- and right- neighbors to one another. Thus we find that at rapid reproduction rate in the *presence* of apoptosis, holes, or cribra, form in areas of cell death, producing the pattern shown in Fig. 1B. On the other hand, at a lower reproductive rate in the *absence* of apoptosis, we find that cribra can again be formed, but in this case due to the merging of papillary fronds into so-called “Roman Arches” [41]. In the first case, cellular reproduction must be very rapid (to produce cell death through overcrowding), while in the second, reproduction can be slower due to the lack of cell death, allowing arches to form. Clearly the speed of reproduction and cell death are important to prognosis, yet these properties are not apparent in 2D sections of tissue specimens.

Based on these observations, we propose that there may be two different mechanisms for cribra formation: one driven by merging papillae leading to an open, branch-like structure, and a second driven by apoptosis leading to a closed, sponge-like, architecture [40], [41]. In support of this proposition, we note that prior work has identified luminal spaces in papilloma and papillomatosis as being a network structure having a ‘slit-like’ appearance, possibly associated with merging papillae, whereas a second, cribriform structure, was identified as being ‘porous’, such that the microlumens have a ‘punched-out’ appearance [33].

The goal of the present paper is to establish whether such distinct morphologies were present in actual 3D reconstructions and the following sections show that they are indeed present. With this aim, we develop tools to analyze 3D morphological characteristics of *ex vivo* DCIS specimens and as we will describe, we find that analysis of the third dimension yields insights into DCIS that are not apparent in 2D. Specifically, we investigated whether the 3D architecture of DCIS exhibits subtypes that cannot be characterized in 2D, with the assumption that these subtypes may relate to different mechanisms of growth.

We clarify that the purpose of this work was not to classify the specimens into carcinoma vs. non-carcinoma, but to use the 3D reconstructions as a tool to evaluate the morphology of the DCIS microluminal structures, and to shed light on the mechanisms of formation leading to an observed state. We found that there were at least two different 3D architectures, ‘bubbles’ and ‘tubes’, identified in the cribriform specimens that were classified as the same 2D subtype. We hypothesize that these architectures relate to the two proposed mechanisms of growth discussed in the previous paragraphs: merging papillae and apoptosis.

## Materials and Methods

### I. Materials

Preliminary data was obtained from the Cancer Institute of New Jersey (CINJ), from which we developed preliminary reconstructions using manual segmentations and itk-snap [42] for developing the 3D reconstruction. These preliminary specimens were unpublished data from biopsy specimens that consisted of up to 100 serial segmented and light microscopy imaged slides (using 4× magnification).

The specimens used for this study were collected from the Tokyo Woman's Medical Hospital from 8 patients. Each specimen was diagnosed with cribriform-type ductal carcinoma *in situ*, serially sectioned into sequential slices 4 microns thick, stained with hematoxylin and eosin (H&E), and mounted on slides. This procedure resulted in 100 serial segmented slides in which 33 ducts were identified and imaged using light microscopy (4× magnification). These slides were imaged using an Olympus DP71 camera, creating images of size 4080 by 3072.

Each specimen could contain numerous ducts, so within these specimens we chose ducts of interest (i.e. a cribriform duct wholly contained within the specimen.) We cropped the image by manually selecting a square around the duct of interest, such that the cropped image ranged from 500 by 500 to 1500 by 1500 pixels^2^ depending on the size of the ductal region of interest. In some datasets, the ductal region of interest reduced in size and closed off before the end of the specimen: in these datasets we used fewer than 100 images. The resulting tiff images are 8 bit red/green/blue (RGB) with a pixel size of approximately 0.9 microns.

From our collected data, we identified in total 18 duct samples that were histologically classified as DCIS (through consultation with Drs. Barnard & Fujibayashi), of good quality, and were at least 40 serial sections in length. Out of these 18, three were not reconstructed because there were alignment issues.

#### Ethics Statement

All samples from CINJ were de-identified, already collected in tissue banks, and obtained through an Internal Review Board (IRB)-approved protocol at CINJ under Internal Review Board: UMDNJ-New Brunswick/Piscataway Campus; Individual consent was not obtained as this was an Exempt (#4) study.

Each dataset from the Tokyo Women's Medical University Medical Center East consisted of de-identified human breast specimens from resected material that were taken for the purpose of therapy. All patients chose for the surgery to be performed. All the data sets were anonymous and only encoded numbers were used to specify the sample. All samples were already collected in tissue banks, and are therefore considered exempt under IRB status. When these specimens were taken, consent was not obtained to use these specimens for research, as this was not standard practice at the time. For de-identified tissues, it was also not standard practice for physicians to obtain approval of IRB from the university to use them for research and publishing and thus was not obtained. We have complied with the ethical standards of Tokyo Women's Medical University Hospital.

### II. Procedure

In order to produce automated 3D reconstructions, we incorporated three tools: 1) an automatic alignment method, 2) an automated segmentation method of a) individual ducts, and b) intraductal cribra, and 3) a 3D reconstruction tool to produce a final 3D representation. We describe each tool in turn below.

#### 1) Automatic Alignment

The first step of our analysis was to automatically register, i.e. align, previously acquired serial images of specimens with DCIS. Registration of slide-mounted sections presents numerous challenges [43]. First, the process of embedding tissues in paraffin prior to slicing causes distortion and shrinkage [43] - a problem that is especially troublesome in breast tissue due to its fatty nature [44]. Additionally, successive images suffer mechanical deformations during slicing and mounting, and cell locations, empty spaces, and fatty tissue differ between consecutive images. Lastly, coloration of the tissue is variable, due both to the necessity of individually staining each section, and to variations associated with features including nuclei, fat, and blood vessels, as well as unwanted debris. We have constructed alignment and segmentation methods that successfully cope with all of these issues to align and segment serial sections from a paraffin-embedded DCIS tissue block.

**Figure 2:**
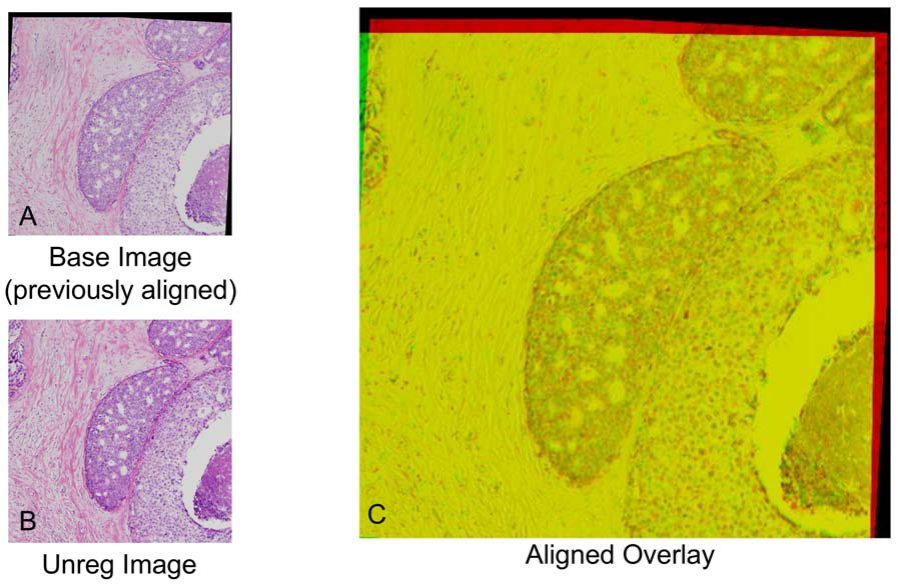
Alignment Results (Sample dataset). **A** “Base” image: 5^th^ slice in sequence of paraffin-embedded exemplar specimen. The image has been previously aligned by comparison with 4^th^ slice, as shown by comparison with black background. **B** “Unregistered” image: This is the 6^th^ sequential slice from the same exemplar specimen. **C** Overlay comparison of base and registered image - formed by rotating and translating the unregistered image as described in text. The base image is shown in red, the registered image is shown in green. Most of the image is correctly aligned, as seen by the mostly yellow (red+green) overlay, and black again represents extra background introduced by alignment.

The alignment method used for this project is “rigid,” meaning it incorporates only rotation and translation, and does not morph or otherwise distort the image, see Fig. 2. The algorithm, implemented in Matlab, is as follows.

- First, we temporarily reduce the resolution of the photomicrographs of adjacent sections to produce images close to 500×500 in size. This serves two purposes: 1) it produces a uniformly successful algorithm that reduces the effects of size variations between images and 2) it improves processing time.
- Second, terming one image the “base” image, and its adjacent image the “unregistered” image, we construct a 150×150 “template” for each image that we will use to compute optimal translation distances and rotation angles. Using a very small size template speeds up analysis and additionally eliminates problems such as small noise features or duct border details.
- To create the template, we perform Otsu thresholding [45] in the red channel (a strong signal for H&E staining). We calculate the centroid of the largest object using the centroid to identify our object of interest, in this case the duct. We construct the 150×150 template using the centroid as the center position.
- We then convert the low resolution template and unregistered images to grayscale, thus lowering the processing time while keeping intensity information, and we rotate the unregistered image about the centroid to maximize the normalized cross-correlation with the base image [46],
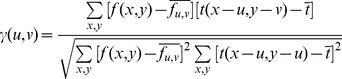
(1)Here, γ(*u,v*) is the normalized cross-correlation, *f*(*x,y*) is the unregistered image, *t*(*x,y*) is the template image, 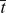 is the mean of the template and 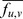 is the mean of *f*(*x,y*) in the region under the template between the low resolution segmented duct base image and rotated unregistered image, *u* and *x* are the row positions whereas *v* and *y* are the column positions of the matrix or image, respectively. Since each image is stained individually, intensities can differ from image to image, and we normalize the cross-correlation to compensate for these differences.
- Then, we rotate the low-resolution unregistered image by an angle that maximizes the cross-correlation, and we fill any new, non-overlapping, areas (see top and right boundaries in Fig. 2) with a background to produce low correlation in these areas.
- After the optimal angle has been calculated, the template is suitably rotated, and then its translation is calculated to similarly maximize the normalized cross-correlation. The maximum translation allowed is 20% of the length of the image in either direction, which is determined by obtaining the best correlation within this range. We find that this restriction effectively prevents occasional false alignments in which the registered image is rotated and translated to align background/stromal areas, leaving the duct areas non-overlapping.
- Finally, we rescale the optimal rotation and translation values obtained using the “template” images to rotate and translate the original high-resolution image.

Once all images have been aligned, segmentation is performed, in two parts: duct segmentation, used to segment the cellular regions from the background (fat and ECM), and intraductal cribra segmentation, used to segment the microluminal spaces within ducts.

#### 2) Automatic Duct Segmentation

**Figure 3:**
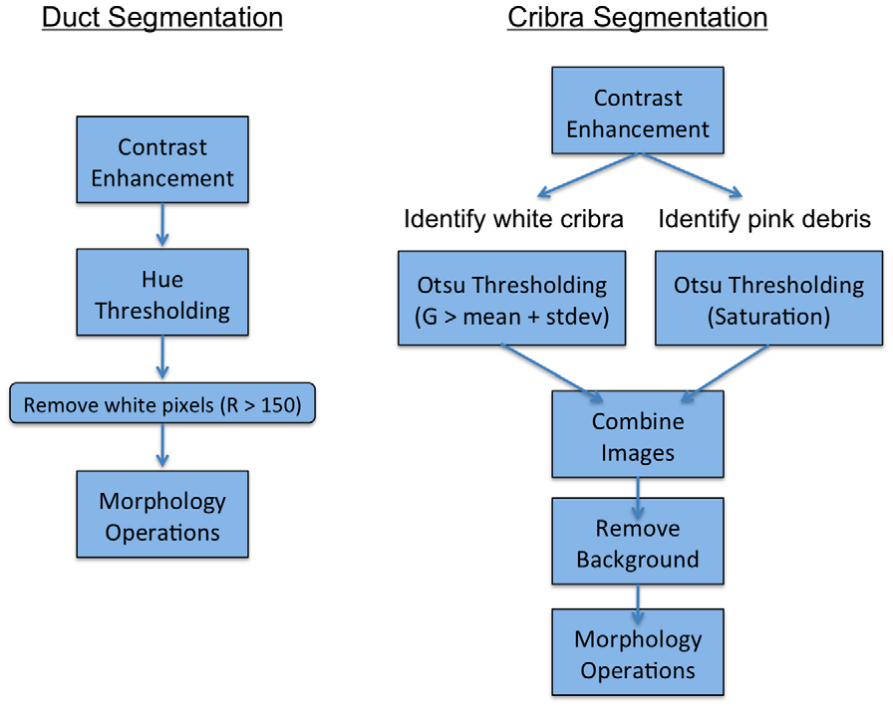
Duct and Cribra Segmentation Flowchart. On the left, we show the general layout of the duct segmentation. We use contrast enhancement to ‘normalize’ each image to account for differences in staining that may occur for each slice. Thresholding is used to binarize the image. Hue segmentation can result in white pixels of the background to be picked up as duct, thus we remove them. Morphology operations are used to smooth the duct segmentations and remove minor artifacts. On the right, we show the layout of the cribra segmentation method. We use contrast enhancement to ‘normalize’ each image and better delineate the cribra. We use thresholding to binarize the image and identify ‘white’ cribra and ‘pink’ debris. These segmentations are combined and the background outside of the duct region is removed. Morphology operations are used to smooth the duct segmentations and remove minor artifacts.

Duct segmentation is performed as follows, see Figure 3. Intensities in the H&E stained images typically range from 100–255, so we first expand these values to span the full range 0–255. This broadens the dynamic range of the image, improving the apparent contrast.

To isolate the ductal regions from the rest of the image, we make use of the fact that H&E staining stains the nuclei of the cells, and thus ductal regions tend to be purple in hue. The red channel alone was sufficient to perform alignment, however we have found that additional information is useful for segmentation. To segment our images, we perform the following steps.

We convert from RGB to HSV, which allows us to more clearly identify purple hues. We define Ir = intensity in the red channel, Ig = intensity in the green channel, and Ib = intensity in the blue channel, and we calculate the Value V = 0.3*Ir+0.59Ig+0.11Ib. Next, we define Cr = Ir−V and Cb = Ib−V, and designate Hue and Saturation according to H = arctan(Cb, Cr) and 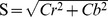, respectively.
We threshold the image using the Hue (H), defined above, so that H<0.7 is mapped to 0, and H≥0.7 become 1, which segments the purple regions; a value determined by trial and error to produce effective segmentation. The latter category selects white as well as purple regions, so we remove white from the segmentation by excluding pixels with red intensities greater than 150.
Next, we perform mathematical morphology operations: opening using a disk of radius 1 and closing using a disk of radius 3, to remove false connections and small protrusions and to close small gaps, respectively. We fill in holes within the segmentation so that the segmented regions are solid. DCIS cell sizes tend to vary but are approximately 10 microns in length. Any pieces that are smaller than the area of six nuclei (2290 square pixels) are removed from the segmentation because these segments are too small to be ducts and are likely noise or isolated cells. We improve the convexity of the segmentation by doing a large closing operation using a disk of radius 9, dilating and eroding each section, smoothing and filling any indentations on the borders of the segmentation. We then fill any holes formed during this process.

#### 3) Automatic Cribra Segmentation

To segment intraductal cribra, we must distinguish true cribra from other ductal features. For example, uneven staining can skew cribra segmentation to make cribra appear larger or more numerous than in actuality. Our cribra segmentation algorithms therefore separately address robust equalization of intensities and morphological operations, which we discuss in turn.

Robust Equalization is used to equalize image features in the presence of unavoidable dye and lighting differences; we perform several steps.

First, we perform histogram equalization [47] with 16 tiles (4×4), using the green channel of the original image. Histogram equalization is a contrast enhancement technique that adjusts the histograms in each tile to approximately match a uniform distribution. This method corrects for uneven staining and increases the contrast in the image, both of which delineate the cribra from the rest of the duct. The green channel is used for this because empirical tests using various channels and combinations reveal that this produces the most veridical cribra segmentation.
Second, we threshold intensity values of the equalized image to identify cribra boundaries. We find again that the green channel discriminates these images best: explicitly, we identify green pixel values over 1 standard deviation above the mean, and use Otsu thresholding [45] to find the threshold value that best delineates this region, defining the cribra to consist of the higher intensity regions. We restrict thresholding to be performed only in a high intensity range, because green intensity histograms exhibit a peak that correlates strongly with cribra locations. As a practical matter, in some images the Otsu threshold value is found to be greater than 242, and in this case we find that using 242 as the threshold produces reliable segmentation.
Third, we find that using just the green channel, our algorithm can erroneously fail to identify a cribrum if it contains cellular debris. This debris is often stained pink, and can be identified by having a high saturation. We therefore correct for debris by normalizing saturation values before performing Otsu thresholding to binarize the image.
Fourth, we combine the Ductal and Cribra segmentations, remove the background areas (outside of the duct), and fill holes identified in the previous step as being white (microlumenal) or pink (microlumenal with debris).
Finally, we erode the segmentation using a disk of radius 4 to correct for minor enlargements of cribra size produced by thresholding and segmentation.

Mathematical morphology operations are then performed to correct for a number of minor errors encountered during algorithm testing. These are as follows.

**Figure 4:**
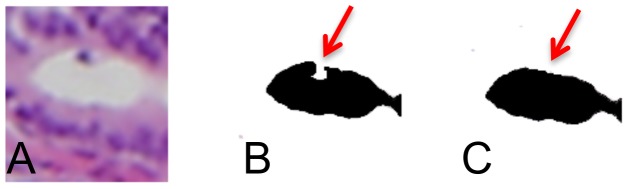
Crescent Issue. **A** An example cribra compared with an illustrative **B** cribra segmentation. Here is an example of the crescent issue (red arrow) that can occur before a closing operation, where the cribra is not a smooth surface but has a crescent-like gap. **C** This shows an illustration of how the closing operation can fix this type of issue.

Debris near the edge of a cribra can produce a cribra with a crescent- rather than a circular- shape, see Figure 4. To correct for this error, we close small gaps by using a closing operation: dilatation followed by erosion, on the image using a disk structuring element of radius 3.
Although most debris tends to occur on the edge of a cribra - and can be removed as described in our segmentation corrections - debris within a cribrum can sometimes be within the cribra itself, in which case it will leave a small hole in the segmentation. We therefore fill in any holes, which appears to correct for this anomaly.
We also find that the edges of our segmentations may not be smooth and may exhibit small protrusions. Likewise, when we fill in small holes, we sometimes produce uneven edges. We therefore smooth segmentation boundaries by using an opening operation: erosion and then dilation, on the image using the same disk size of radius 4. The closing operation performed here can close small gaps leaving a connected edge with a small hole at the location of the gap. Therefore we fill in any holes a second time to correct for this error.
Finally, cribra on borders of the image may not be completely imaged, so we clear the vicinity of borders to remove these incomplete cribra. We remove small objects (<100 pixels^2^) since they are too small to be considered cribra.

#### 4) 3D Reconstruction

To produce a 3D reconstruction, we stack the binary images obtained from the cribra segmentation algorithm. We reduce the size of the images so that 1 voxel is approximately 4 microns in a side, since each serial section is 4 microns thick. We create surface points in the volume data using the Matlab function ‘isosurface,’ which produces surfaces from sequential closed curves, and we connect the surface vertices using the Matlab ‘patch’ function [48]. This produces a 3D structure, which we smooth using Matlab's ‘isonormals’ function.

We hypothesized that there would be two types of cribra present in the 3D reconstructions, ‘bubble-like’ cribra resembling small holes throughout the duct or ‘tube-like’ cribra that ran most of the way through the duct. The length of the duct ranged from 40 sections to 99 sections and we assumed the size of ‘bubble-like’ cribra should be small irrespective of the length of the duct. Thus, the 3D reconstructions were classified as ‘bubble-like’ if the largest cribrum was <25 sections (100 microns) in height, ‘tube-like’ if the largest cribrum was >35 (140 microns), and ‘unclear’ if it was between these two values.

### III. Statistics

To evaluate the accuracy of our automatic segmentation algorithms, we manually segmented 5 slices from each of 20 different serially sectioned duct datasets, which resulted in a dataset of 100 manually segmented cribra images. We compared the manually segmented images to the automatically segmented cribra using “precision” and “recall”, two of many possible statistical metrics available [49]. Precision and recall (Eq's 2,3) are commonly used in engineering and segmentation research [50], [51], and provide objective measures of how well an imaging algorithm agrees with “ground truth.” In general terms, precision is a measure of how many pixels chosen by the algorithm are outside of the lesion (false positives: FP) as compared with correctly identified lesion pixels (true positives: TP). By contrast, recall is a measure of how many lesion pixels are missed by the algorithm (false negatives: FN) as compared with true lesion pixels. In this way, precision and recall balance one other and often a high score in one implies a low score in the other. Finally, ground truth is defined to be images that are manually segmented in consultation with a practicing cancer pathologist (Dr. N. Barnard).

Precision and recall are defined as follows:
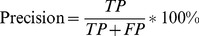
(2)

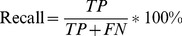
(3)In order to assess the accuracy of the 3D reconstructions, we built 3D reconstructions using the automatic reconstruction method and manual reconstructions and verified that the basic architectures were the same. We also plot the heights vs. aspect ratio of a pair of representative reconstructions. We use linear fitting of the data using least squares, with the polyfit() function in Matlab.

## Results

To demonstrate the feasibility of our approach for building 3D reconstructions, first we examined the performance of the alignment. We found that the alignment method was successful for most of the images (there was a 3.6% error rate out of 1712 images that were examined), as in the example case shown in Figure 2. In Fig. 2C, areas of agreement between successive sections appear in yellow, while areas that differ appear in red or green. For the example shown, it is evident that most of the image is yellow and thus the image is aligned nearly everywhere.

**Figure 5:**
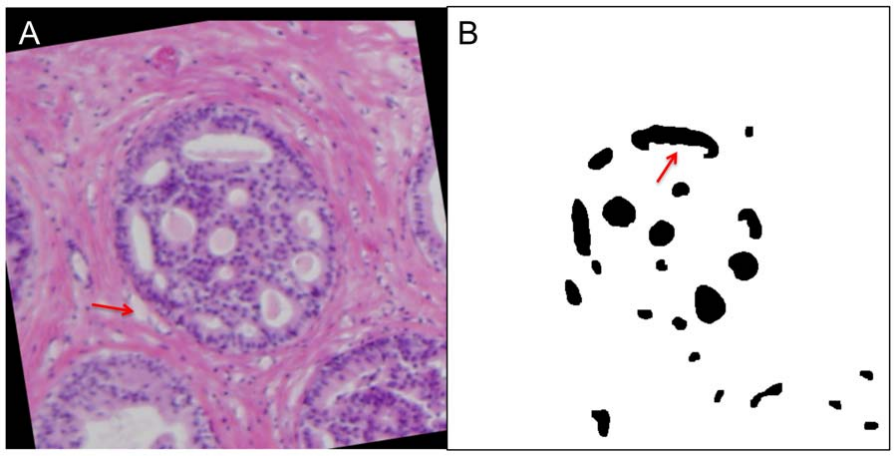
Cribra Segmentation. **A** Original aligned image, compared with **B** automatic cribra segmentation. Most of the cribra are completely identified but in some cases some of the cribra is absent or incomplete, see red arrow in **B**. In other cases there are false cribra but these are small and few in number. In **A** the red arrow indicates a fat globule that can be incorrectly identified as a cribrum. This example has a Precision score of 81.1 and a Recall score of 82.0.

Next, we used our segmentation approach to segment out the ducts and the cribriform regions within the ducts. We seek to determine the 3D architecture of these regions, specifically examining whether they have a closed, ‘bubble-like,’ architecture or an open, ‘tube-like,’ architecture. We show a typical segmentation in Figure 5. In this image the original image has been aligned in Fig. 5A, and the segmented cribra image, Fig. 5B, shows the duct and background as white and the cribra as black. We find that sometimes the duct segmentation algorithm includes areas outside of the actual ductal regions, such as the fat globule in Fig. 5, but that the segmentation algorithm seems to reliably capture cribra.

To assess the accuracy of our automatic cribra segmentation, we compared the manually segmented dataset (100 images) to their respective automatic segmented images. We determined that the median precision of the algorithm is 85.5%, with mean 74.9%, and interquartile range of 23.9; the median recall is 76.3%, with mean 71.2%, and interquartile range of 19.4, see Table 1. In some cases known issues appeared due to a large amount of stained immuno-infiltrate that was recognized as cellular material and thus interfered with the duct segmentations. If we exclude these samples (20) as shown in Table 1 as the “Reduced Dataset”, the reduced dataset achieved a median precision of 86.7%, a mean precision of 80.1% and an interquartile range of 20.4%. The reduced dataset had a median recall of 76.1, a mean recall of 70.6%, and an interquartile range of 19.1%. Based on these findings and the visual results, see Fig. 5, we conclude that our automatic segmentation algorithm is sufficiently accurate to reliably build 3D reconstructions.

**Table 1:** Precision and Recall of Automatic Cribra Segmentation Algorithm.

Automatic Cribra Segmentation	Number of Samples	Precision (Median)	Recall (Median)
Total Dataset	100	85.5%	76.3%
Reduced Dataset	80	86.7%	76.1%

**Figure 6:**
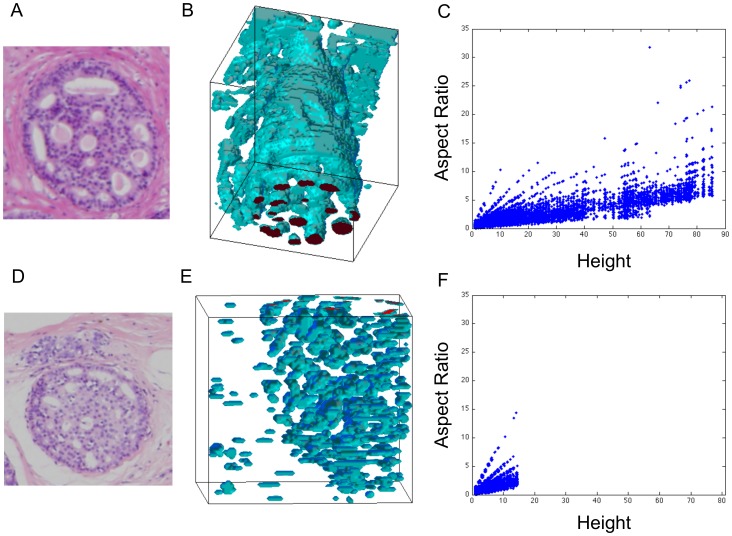
Exemplars of Bubbles and Tubes from Automatic Reconstructions. **A** Example of a serial section showing the duct of interest, taken at 4×. **B** 3D reconstruction (99 sections) of corresponding microlumenal structure showing ‘tube-like’ architecture. **C** Plot of cribra height (in voxels) vs. aspect ratio from the 3D reconstruction. **D** Example of a serial section showing a duct of interest at 4×, note the similarity to panel **A**. **E** 3D reconstruction (80 sections) of corresponding microlumenal structure showing ‘bubble-like’ architecture. **F** Plot of cribra height (in voxels) vs. aspect ratio from the 3D reconstruction of the second specimen, note the clear differences in aspect ratios.

Finally, we assembled successive slices into 3D reconstructions, as described in the Materials and Methods section. We found two types of cribra architectures. First, as shown in Fig. 6A,B&C, we find what we term ‘tubes’ – large and connected cribra that extend the length of the specimen, ∼90, and with aspect ratio up to ∼25. Second, shown in Fig. 6D,E&F, we found what we term ‘bubbles’ – cribra with maximum dimension ∼15 voxels and aspect ratio ≤15: these appear as small, isolated regions that do not extend through the length of the specimen. We emphasize that the 2D sections, shown as insets to Fig's 6A&D, are similar, yet the 3D structures differ significantly.

In Figure 6B&E, we show 3D reconstructions of two exemplar datasets illustrating the qualitative differences between the two architectures found, and in panels C&F, we show quantitative comparisons between the two architectures. For rotated views of these 3D reconstructions see Videos S1 & S2.

The plots in Fig's 6C&F, were produced by sampling 10,000 voxels from each reconstruction. For each voxel within a lumenal space, we evaluate the length, width, and height, and plot for each pixel the aspect ratio (height/length) as a function of height. Length, width, and height are measured in terms of number of voxels, with each voxel approximately being equivalent to 4 microns cubed. Length and width are determined by evaluating the number of contiguous voxels within the lumenal space in the x-, y-, and z- directions, where x and y are in the plane of the slice, and z is the normal to a slice. Voxels outside of lumenal spaces are not included in this calculation. These plots demonstrate that ***essentially indistinguishable 2D sections*** of cribriform DCIS (insets to Fig's 5A&D) exhibit ***3D morphologies that differ both qualitatively and quantitatively***.

### I. Validation

**Figure 7:**
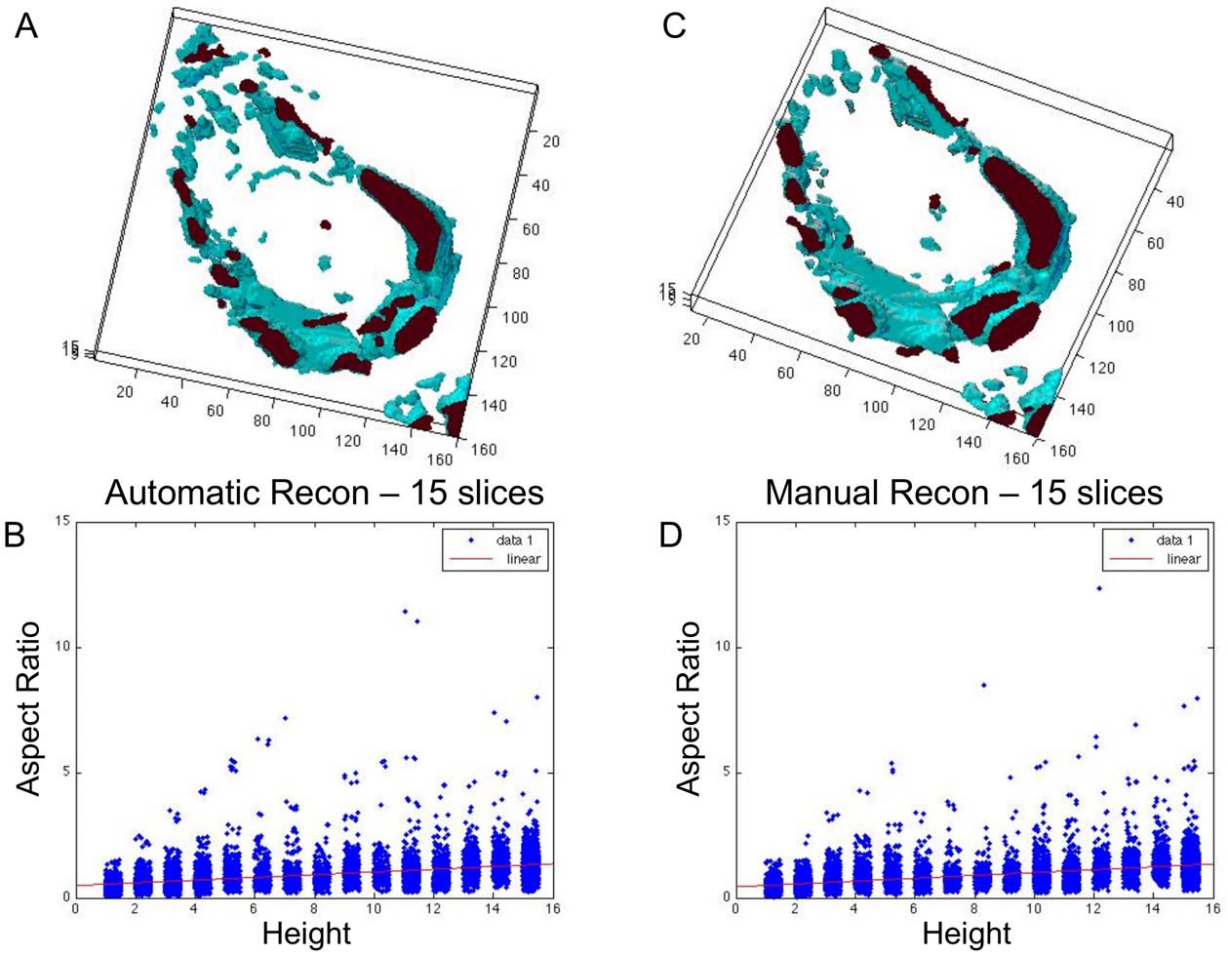
Automatic vs. Manual Segmentation. **A** is the 3D reconstruction of 15 slices using the automatic segmentation algorithm. **B** shows the plot of the aspect ratio vs. height. **C** is the 3D reconstruction of 15 slices using manual segmentations. **D** shows the plot of the aspect ratio vs. the height for the manual reconstruction. Both quantitatively and visually the architectures of the reconstructions are consistent. The axes and heights are in voxels.

To verify that the 3D reconstructions accurately represent the 3D architectures of the cribra, we manually segmented cribra in 15 serial sections from 5 different ducts, resulting in a total dataset of 75 manually segmented images. We built a 3D reconstruction, using the same method described above, of the manually segmented cribra and compared it to the automatically segmented 3D reconstruction. A typical comparison is shown in Figure 7, once again the axes and heights are in voxels. In the figure, we also plot the aspect ratio vs. the height for both reconstructions and find them to be nearly identical. We used a linear fitting algorithm for each reconstruction and found the linear fits to be similar. The automatic reconstruction data had a linear fit of y = 0.05x+0.50 with a norm of residuals 57.6. The manual reconstruction had a linear fit of y = 0.06x+0.45 with a norm of residuals 66.5. Therefore, we conclude that the automatic 3D reconstructions are accurate enough to describe the 3D morphology of the cribra.

As explained in the materials and methods section, 15 specimens remained in the dataset that fit the inclusion criteria. These 15 datasets were reconstructed and classified, see Table 2. Twelve out of the 15 specimens that we examined exhibited 3D reconstructions that fit into two categories of patterns: isolated bubbles scattered throughout most of the ductal region, or a network of cribra located near the duct borders. We found 3 cases in which the 3D reconstructions fell into the intermediate category where it was unclear which class it belonged to. There were samples in which both types of structures seemed to appear as well.

**Table 2:** Breakdown of ‘Bubble-like’ and ‘Tube-like’ 3D Reconstructions.

‘Bubble-like’	‘Tube-like’	‘Unclear’	Total
6	6	3	15

## Discussion

To summarize, we have developed an approach to reconstruct 3D images of cribriform ductal carcinoma in situ (DCIS). The approach consists of four parts, 1) alignment, 2) duct segmentation, 3) cribra segmentation, and 4) 3D reconstruction. We have evaluated the overall accuracy of our approach in terms of precision and recall and achieved good accuracy. Since our work is less dependent upon completely segmenting the entire cribra and more upon accurately characterizing the 3D morphology, in particular whether the cribra are connected or separate, having 90% accuracy was not necessary. Even if the entire cribra was not segmented in a section, the piece that was segmented would still connect to the overlapping piece in the successive cribra, and therefore the 3D morphology would be the same. We have also independently assessed the accuracy of the 3D reconstructions and found that the essential features of the 3D cribra architectures were consistent for the automatic and manual reconstructions.

Importantly, from our analysis we have identified for the first time that two distinct categories of 3D cribriform structures are present in human tissue samples. First, we have found ‘bubble-like’ cribra, suggestive of a possible apoptotic mechanism that may destroy localized overpopulated regions, leaving behind empty spaces. In these specimens, we found cribra scattered throughout the duct without any evident pattern. Second, we found ‘tube-like’ cribra that extend through the length of the specimen, consistent with the possibility that these cribra may have been formed through the documented mechanism of merging of micropapillary fronds [41]. In this case, we saw that the cribra seemed to be located toward the outside of the duct, paralleling the ductal architecture, with very few cribra within the duct interior. Other work has identified these two types of microluminal spaces, but in that work, it was reported that interconnected lumens were found in papillomatosis, whereas the round isolated ‘bubble-like’ lumina were found in cribriform DCIS [33]. All of our samples, by contrast, have been identified by an experienced cancer pathologist (NB) to be cribriform in structure.

The identification of two distinct types of 3D architectures in cribriform DCIS supports the hypothesis that these are the result of differing mechanisms governing growth and progression of the disease. These mechanisms of growth may lead to different patient outcomes, and thus have prognostic value. For instance, if as predicted from our previous model [40], ‘bubble-like’ architectures are the result of cells with high proliferation rates and apoptosis and ‘tube-like’ are the result of cells with moderate proliferation and low apoptosis, this would suggest that distinct pathways are affected in each case. DCIS displaying ‘bubble-like’ architectures would most likely have developed self-sufficiency in growth signals or resistance to anti-growth signals, such as through alterations in the Ras or pRb pathways [52]. In contrast, DCIS displaying ‘tube-like’ architectures would most likely have developed resistance to apoptosis, such as through alterations in p53 [52]. Thus these architectures may not only reflect differences in the mechanism of progression but also differences in the cellular properties, which could correspond to different patient outcomes. We propose that some of the difficulties in determining prognosis for DCIS lesions may be a result of this heterogeneity that is not reflected in the current 2D analysis of lesions and further investigation is warranted.

It is clear from this research that image segmentation and 3D reconstructions are valuable tools that promise to benefit breast cancer and other areas of cancer research. For instance, Bartels, Thompson, and colleagues, developed an automated segmentation and prognosis system for histopathology [27]. Such techniques have been developed for prostate [27], [28], colorectal [29], and ductal carcinoma *in situ* (DCIS) tumor tissue [30]. For DCIS, their method focuses on segmenting out the ductal and luminal region based on a previous method for segmenting cribriform gland tissue of the prostate [53]. The segmentation approach uses a number of thresholding techniques, morphometric operations, and the use of a knowledge file [54] to segment out the ductal structures [30]. A cribriformity index was also developed as a measure of the ratio of microlumens to gland area within a duct [30]. They were able to distinguish DCIS from ductal hyperplasia using their segmentation method, but were unable to segment 14% of the images. A number of image segmentation techniques have also been applied for automated border detection in dermoscopy images (see [55] for a review).

Imaging and 3D reconstruction techniques can also be useful tools for visualizing the spread of pre-invasive, invasive and/or metastatic breast cancer within the ductal system. For instance, the size or extent of DCIS is often measured from a single slide, which is known to be inaccurate and to underestimate extent [56], [57]. Many existing methods, such as using calcifications, mammography, and blocks to measure extent have also been shown to underestimate the extent of the disease [56]–[59]. The improvement of imaging and reconstruction techniques may provide the necessary tools for developing an accurate way of measuring extend. 3D reconstructions from serial sections, such as we have described, may be a useful tool for visualizing invasive and metastatic disease as well. In fact, the spatial arrangement of areas of normal and cancerous breast tissue were distinguishable using sequential sections and a tumor marker, c-erbB-2 [60]. Using 3D reconstructions of sentinel lymph nodes, another study developed 3D visualizations of metastatic breast cancer and found that metastatic growth occurred in three patterns: sinusoidal, nodular, and diffuse [61]. With improvements in imaging techniques, the ability to visualize the spread of individual patient's breast cancer certainly has the potential to improve diagnosis and early detection.

Lastly, it seems reasonable to expect that other cancers – for example prostate or colorectal cancer – may likewise exhibit 3D morphologies that present a record of the details of past growth and future prognosis not available from 2D sections. 3D co-cultures and dermoscopy demonstrate that 3D information could help in distinguishing non-malignant from malignant phenotypes [13], [62]. The morphology of many different cancers has been found to be different in 2D vs. 3D and the importance of 3D cell models has already been established, see [63]. Thus, we propose that the examination of the 3D morphology of human cancers could be the next step in understanding cancer progression and improving cancer diagnosis.

In conclusion, two different 3D cribriform architectures were identified that would not be identifiable using traditional 2D visualizations. The differing architectures may be governed by different mechanisms, and therefore may ultimately have different progression or invasive potentials. It seems plausible that these different architectures represent different histories of cellular growth, and consequently it would seem prudent in the future to determine whether 3D reconstructions may be a useful tool for diagnostic - and prognostic - evaluations of DCIS.

A future direction of this work would be to produce a robust 3D in silico biomechanical simulation of DCIS progression that we could correlate with the 3D reconstructions. Since we hypothesize that 3D biomechanical interactions between cells play a central role in selection of which of the four known morphologies of DCIS appear, and we have shown that the 3D morphology of DCIS is complex, extending the 2D model to 3D would help further elucidate the progression of DCIS. In addition, such a simulation could explain the conditions that lead to bubbles vs. tube formation of cribra, which would be validated using the 3D reconstructions. We speculate that these distinct growth patterns may lead to different patient outcomes.

## Supporting Information

Video S1 Movie of a ‘Tube-like’ 3D Reconstruction.
(MOV)

Video S2 Movie of a ‘Bubble-like’ 3D Reconstruction.
(MOV)
